# Trends and predictors of laparoscopic bilateral inguinal hernia repair in Spain: a population-based study

**DOI:** 10.1007/s00464-023-09967-y

**Published:** 2023-03-13

**Authors:** Nils Jimmy Hidalgo, Salvador Guillaumes, Irene Bachero, Victor Holguín, Dulce Momblán

**Affiliations:** Department of Gastrointestinal Surgery, Institute of Digestive and Metabolic Diseases, Hospital Clinic, C. de Villarroel, 170, 08036 Barcelona, Spain

**Keywords:** Inguinal hernia repair, Bilateral inguinal hernia, Laparoscopy, Hernioplasty, Perioperative complications, Population-Based study

## Abstract

**Background:**

International guidelines currently recommend laparoscopy for bilateral inguinal hernia repair (BIHR). Our study aims to evaluate the trends and factors associated with the choice of laparoscopy for BIHR in Spain.

**Methods:**

We performed a retrospective analysis of patients undergoing BIHR between 2016 and 2019. We used the national database of the Spanish Ministry of Health: RAE-CMBD. We performed a univariate and multivariable logistic regression analysis to identify the factors associated with the utilization of laparoscopy. We identified perioperative complications and the factors associated with their occurrence through multivariable logistic regression analysis.

**Results:**

A total of 21,795 BIHRs were performed: 84% by open approach and 16% by laparoscopic approach. Laparoscopic approach increased from 12% in 2016 to 23% in 2019 (*p* < 0.001). The 40% of hospitals did not use laparoscopy, and only 8% of the hospitals performed more than 50% of their BIHRs by laparoscopy. The utilization rate of laparoscopy was not related to the number of BIHRs performed per year (*p* = 0.145). The main factor associated with the choice of laparoscopy in multivariable logistic regression analysis was the patient’s region of residence (*OR* 2.04, 95% *CI* 1.88–2.21). Other factors were age < 65 years (*OR* 1.65, 95% *CI* 1.52–1.79) and recurrent inguinal hernia (*OR* 1.31, 95% *CI* 1.15–1.49). The type of approach for BIHR was not independently associated with perioperative complications.

**Conclusions:**

Despite a significant increase in recent years, laparoscopic BIHR in Spain remains low. The main factor associated with the utilization of laparoscopy was the patient’s region of residence; this factor seems to be related to the presence of hospitals with a high rate of laparoscopic approaches where the patient lives. The type of approach was not independently associated with perioperative complications. More efforts are needed to increase laparoscopic use in patients with bilateral inguinal hernias.

**Graphical abstract:**

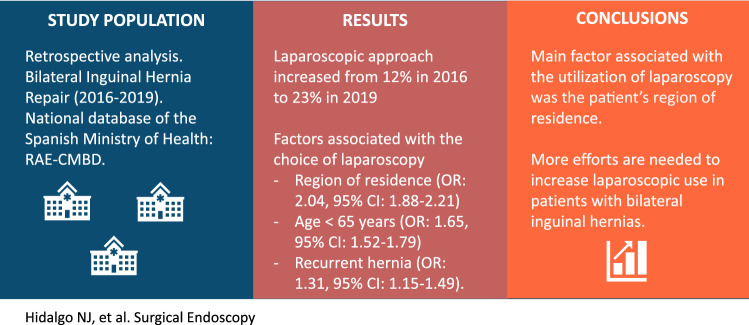

**Supplementary Information:**

The online version contains supplementary material available at 10.1007/s00464-023-09967-y.

Inguinal hernia continues to be an important surgical problem due to its high frequency [1, 2] and socioeconomic consequences, especially in the economically active population. More than 20 million inguinal hernia repairs are performed worldwide yearly [3].

Inguinal hernia surgery has evolved from herniorrhaphy techniques to tension-free techniques with the use of synthetic mesh and, in recent decades, to minimally invasive procedures such as laparoscopic transabdominal preperitoneal (TAPP) [4] and totally extraperitoneal repair (TEP) [5]. The reported benefits of laparoscopic repair include reduced postoperative pain, shorter hospital stay, and shorter recovery [6–10].

International guidelines currently recommend laparoscopic repair for bilateral inguinal hernias [11–15]. The laparoscopic approach is recommended due to its socioeconomic benefits, especially in young patients [13].

However, despite the demonstrated benefits of laparoscopy and international guideline recommendations, laparoscopic inguinal hernia repair has been slow to gain acceptance, perhaps due to problems with surgical technique, learning curve, or cost [16]. The reported use of laparoscopy in inguinal hernia repair is variable: 40% in the USA [17, 18], 23% in England [19], and 5.7% in Spain [20]. Many studies have been conducted assessing the use and results of laparoscopic surgery in inguinal hernia. However, few studies specifically assess bilateral inguinal hernia repair, and results are often mixed with those for unilateral hernia.

Our study aims to evaluate the trends and factors associated with the choice of laparoscopy for bilateral inguinal hernia repair (BIHR) in Spain. Secondarily, it aims to evaluate perioperative complications and their association with the approach choice.

## Materials and methods

### Study design

We conducted a retrospective observational study using the Hospital Discharge Registry of the Spanish Ministry of Health (Registro de Actividad de Atención Especializada-Conjunto Mínimo Básico de Datos, RAE-CMBD) [21]. The RAE-CMBD collects the healthcare activity of all public and private hospitals in the country.

Since 2016, the RAE-CMDB has collected 20 diagnoses and 20 procedures for each patient based on the International Classification of Diseases Version 10 (ICD-10).

### Study population

The study population includes patients who underwent a bilateral inguinal hernia repair procedure in the Spanish National Health System hospitals from 2016 to 2019.

Inclusion criteria: (1) Patients with primary and recurrent bilateral inguinal hernia repair procedure and(2) Age over 14 years.

Exclusion criteria: (1) Manual reduction of the hernia.

**Fig. 1:**
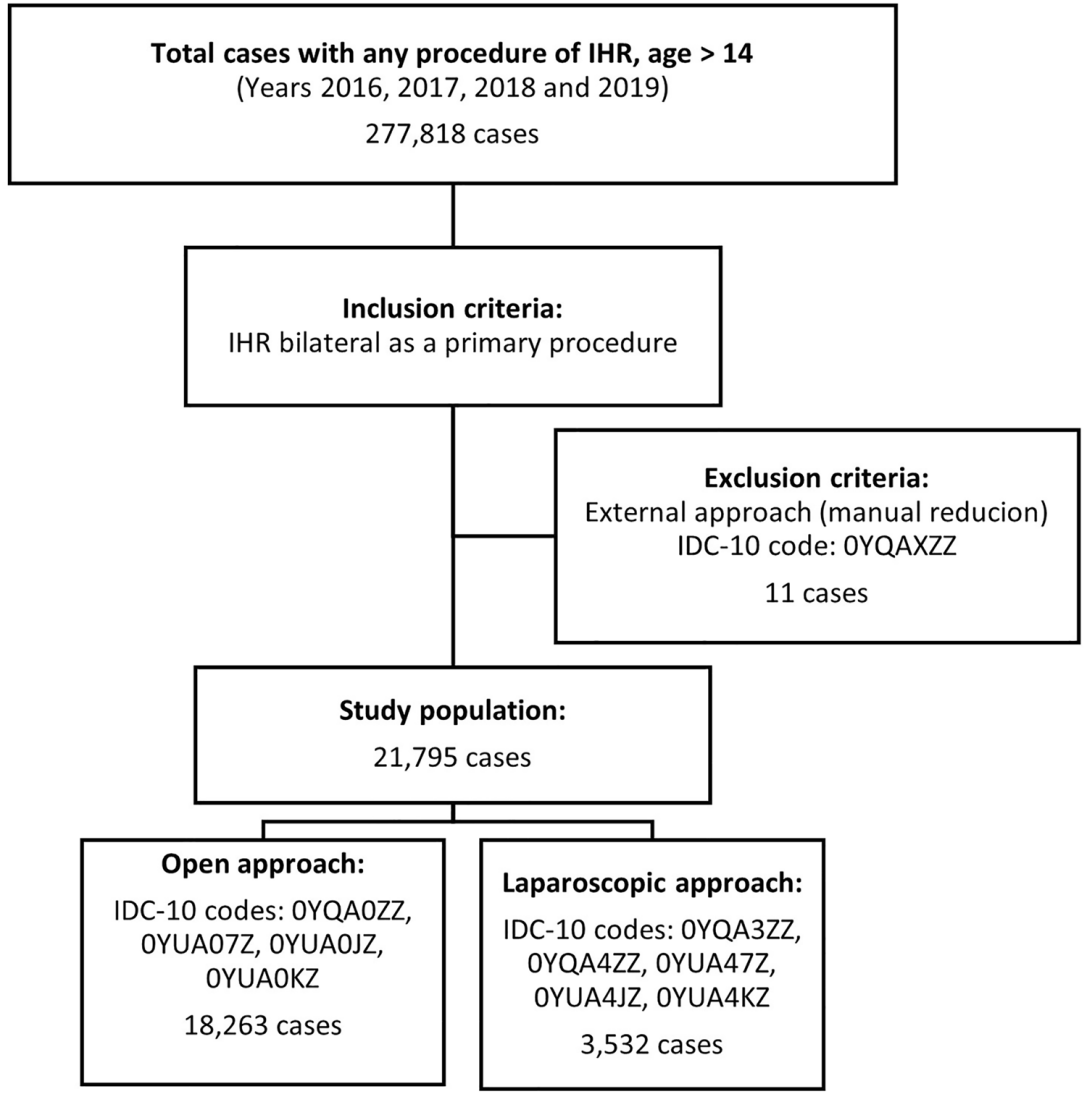
Case Selection Flow Chart IHR: Inguinal hernia repair IDC-10: 10th revision of the International Statistical Classification of Diseases

The flowchart (Fig. 1) shows the ICD-10 diagnosis codes used to identify patients.

### Variables analyzed

#### Demographic characteristics and comorbidities

Demographic and clinical data included age, sex, and region of residence. We identified comorbidities at hospital admission for each patient. To identify specific comorbidities, we used the ICD-10 diagnosis codes described by Quan et al. [22]. ICD-10 codes for comorbidities are presented in the supplementary material.

We analyzed the rate of utilization of laparoscopy according to geographic distribution. Spain is divided into 19 territorial entities called Autonomous Communities, the first political and administrative division level.

#### Hospital characteristics

We analyzed the distribution of high-utilization hospitals (defined as laparoscopy utilization in BIHR of 50% or higher) in Autonomous Communities. The laparoscopy utilization rate by Hospital Volume (defined as BIHR performed in one year) was also analyzed.

#### Characteristics of the hernia and surgery

The hernia characteristics collected were recurrence and complicated hernia (obstruction or gangrene). The surgical approach used was described as open or laparoscopic. In addition, the type of admission for surgery was identified as inpatient o outpatient.

#### Outcomes and perioperative complications

The variables analyzed were hospital stay and perioperative complications such as bleeding, hematoma and seroma, pulmonary complication, cardiac complication, renal complication, urinary retention and infection, paralytic ileus, and visceral and vascular injuries. We used the POA-N (not present at admission) indicator to identify perioperative complications during hospitalization. ICD-10 codes for perioperative complications are presented in the supplementary material.

### Statistical analysis

The Cochran-Armitage trend test was used to evaluate the presence of a statistically significant trend associated with the choice of laparoscopic access during the years evaluated.

The χ^2^ test was used to analyze qualitative variables. For normal distributions, quantitative variables were compared using Student's t-test for two groups, and the non-parametric test used was the Mann–Whitney U-test.

We performed a multivariable logistic regression analysis to identify factors associated with the choice of laparoscopy. Perioperative complications of BIHR were also evaluated, and associated factors were identified by multivariable logistic regression analysis.

Statistical analyses were performed with IBM SPSS 20.0 (IBM Corp. in Armonk, NY). Statistical significance was set at *p* < 0.05.

### Ethical aspects

All the data analyzed are anonymous and were extracted from the database managed by the Spanish Ministry of Health. It is impossible to identify the patients at the individual or reporting unit level under the Spanish legislation. Therefore, this study did not require approval from a Medical Research Ethics Committee.

## Results

### Trends of the laparoscopic approach

Our study included 21,795 bilateral inguinal hernia repairs. During the analyzed period, 18,263 (84%) surgeries were performed by open approach and 3,532 (16%) by laparoscopic approach.

**Fig. 2:**
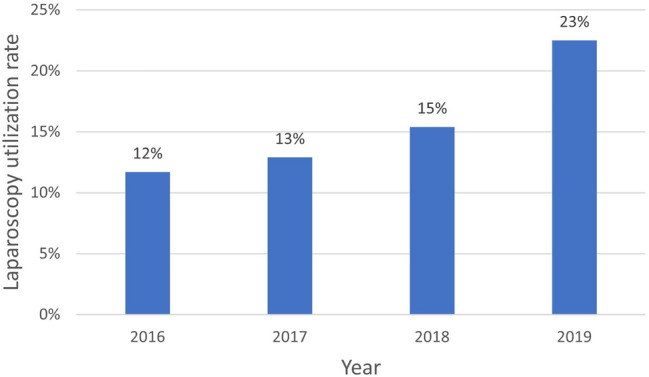
Laparoscopy utilization rate in Bilateral inguinal hernia repair. Cochran-Armitage test for trend was significant (*p* < 0.001)

We observed an increase in the choice of laparoscopic approach during the study period (2016: 12%, 2017: 13%, 2018: 15%, 2019: 23%) and found a significant trend (*p* < 0.001) in the Cochran-Armitage test (Fig. 2).

### Use of the laparoscopic approach in Spanish hospital

**Fig. 3:**
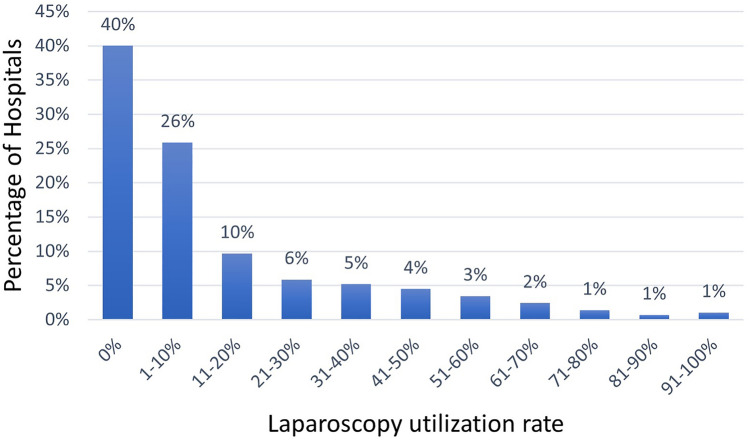
Percentage of Hospitals by laparoscopy utilization rate in bilateral inguinal hernia repair

When we examined the use of the laparoscopic approach for BIHR in Spanish hospitals, we found that 40 percent of hospitals did not use laparoscopy. Furthermore, only 8% of the hospitals performed more than 50% of their BIHRs by laparoscopy (Fig. 3).

### Distribution of laparoscopic high-utilization hospitals

The distribution of high laparoscopic utilization hospitals (50% or higher) was unequal across the country. The regions with the higher percentage of high-utilization hospitals had, in general, the highest use of laparoscopy for BIHR. (Table 1). Only in 4 of 19 regions (Autonomous Communities) did the laparoscopic BIHR exceed 20%.

**Table 1:** Bilateral inguinal hernia repair by region of residence (2016–2019)

Autonomous communities	Total N	Open *N* (%)	Laparoscopic *N* (%)	High-utilization hospitals (%)
Andalucía	2529	2078 (82)	451 (18)	9
Aragón	1027	974 (95)	53 (5)	0
Asturias	548	428 (78)	120 (22)	20
Balears	214	198 (92)	16 (8)	0
Canarias	373	305 (82)	68 (18)	0
Cantabria	315	308 (98)	7 (2)	0
Castilla y León	1591	1295 (81)	296 (19)	7
Castilla-La Mancha	1000	987 (99)	13 (1)	0
Cataluña	2380	1725 (72)	655 (28)	19
Comunidad Valenciana	2314	1861 (80)	453 (20)	14
Extremadura	411	403 (98)	8 (2)	0
Galicia	1494	1237 (83)	257 (17)	13
Madrid	4165	3394 (81)	771 (19)	13
Murcia	700	660 (94)	40 (6)	0
Navarra	727	719 (99)	8 (1)	0
Pais Vasco	1900	1631 (86)	269 (14)	7
Rioja	101	55 (54)	46 (46)	50
Ceuta	3	3 (100)	0	0
Melilla	3	2 (67)	1 (33)	0
Total	21,795	18,263 (84)	3532 (16)	10

High-utilization hospitals: proportion of hospitals who utilized laparoscopy in ≥ 50% of their cases

### Hospital volume

**Fig. 4:**
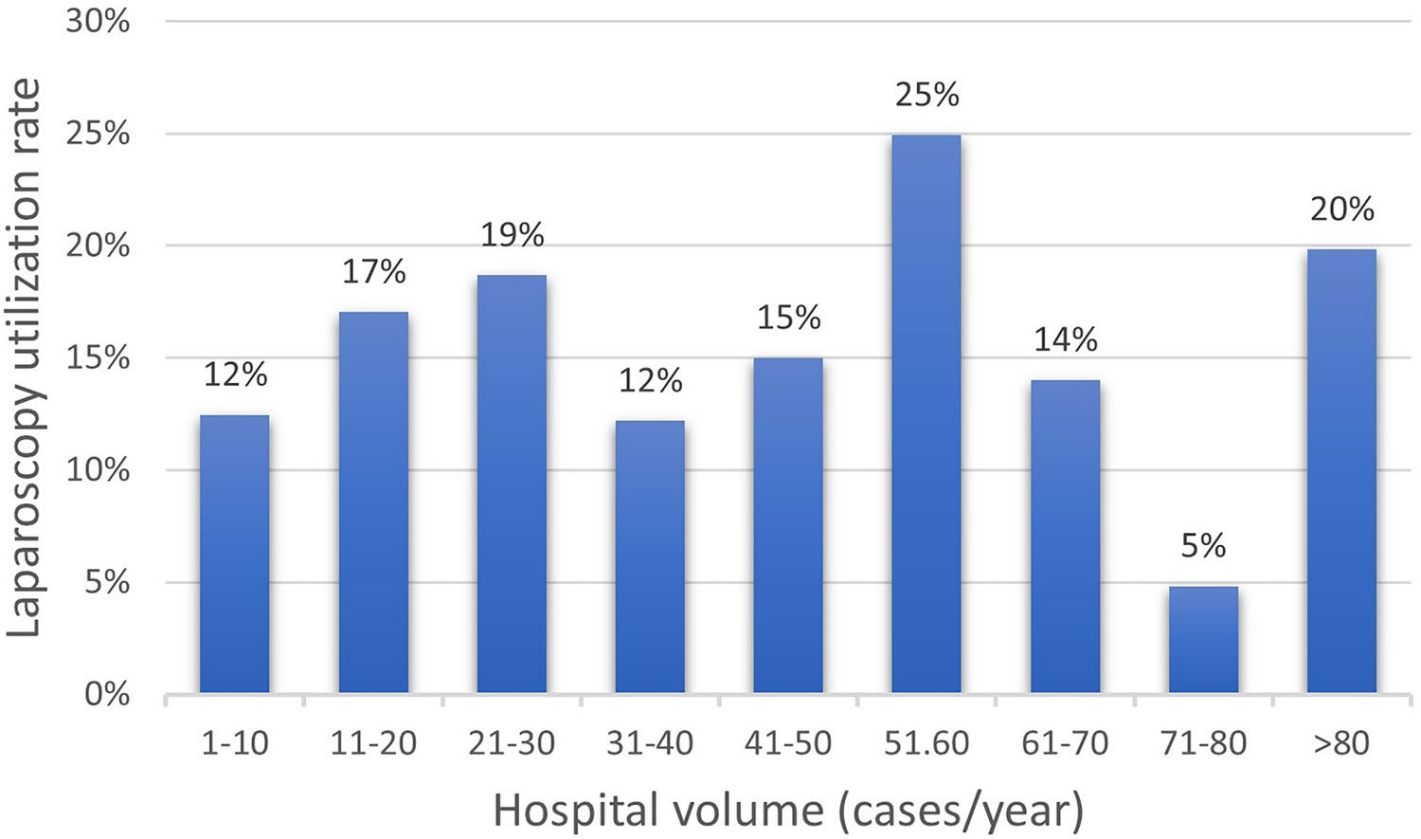
The utilization rate of laparoscopy in bilateral inguinal hernia repair by hospital volume. Cochran-Armitage test for trend was no significant (*p* = 0.145)

The utilization rate of laparoscopy was not proportionally higher in hospitals with a greater number of BIHR (open and laparoscopic) performed per year (*p* = 0.145) (Fig. 4).

### Demographic characteristics and comorbidity

When we analyzed the study population divided into two groups according to the type of approach (open or laparoscopic), we observed that the mean age in the open group was higher than in the laparoscopic group (62.27 ± 13.49 vs. 57.08 ± 13.2, *p* < 0.001) and we found no significant differences in sex (Table 2). The proportion of comorbidities evaluated, except liver disease, was higher in the open surgery group than in the laparoscopic group. The proportion of recurrent hernia in the laparoscopic group was higher than in the open group (8.7% vs. 7.1%, *p* < 0.001). The proportion of outpatient surgery was higher in the laparoscopic group than in the open group (39.5% vs. 32.2%, *p* < 0.001). Hospital stay was longer in the open group (1.2 ± 1.95 days vs. 0.97 ± 1.32 days, *p* < 0.001).

**Table 2:** Characteristics of patients with bilateral inguinal hernia repair (2016–2019)

	Total *N* = 21,795	Open *N* = 18,263	Laparoscopy *N* = 3532	*p* value
Age, Mean ± SD	61.43 ± 13.58	62.27 ± 13.49	57.08 ± 13.2	< 0.001
Age < 65 years, N (%)	12,266 (56.3)	9,863 (54)	2,403 (68)	< 0.001
Age ≥ 65 years, N (%)	9529 (43.7)	8400 (46)	1129 (32)	< 0.001
Sex, N (%)	–	–	–	0.371
Male	20,243 (92.9)	16,950 (92.8)	3293 (93.2)	–
Female	1552 (7.1)	1313 (7.2)	239 (6.8)	–
Comorbidities, N (%)	–	–	–	–
Arterial hypertension	5512 (25.3)	4818 (26.4)	694 (19.6)	< 0.001
Heart disease	1676 (7.7)	1528 (8.4)	148 (4.2)	< 0.001
Chronic pulmonary disease	1277 (5.9)	1122 (6.1)	155 (4.4)	< 0.001
Renal disease	386 (1.8)	366 (2)	20 (0.6)	< 0.001
Liver disease	319 (1.5)	277 (1.5)	42 (1.2)	0.138
Diabetes mellitus	1839 (8.4)	1618 (8.9)	221 (6.3)	< 0.001
Obesity	531 (2.4)	464 (2.5)	67 (1.9)	0.023
Peripheral vascular disease	256 (1.2)	239 (1.3)	17 (0.5)	< 0.001
Cerebrovascular disease	97 (0.4)	89 (0.5)	8 (0.2)	0.033
Rheumatic disease	162 (0.7)	148 (0.8)	14 (0.4)	0.009
Alcohol abuse	421 (1.9)	366 (2)	55 (1.6)	0.077
Tobacco use	2515 (11.5)	2114 (11.6)	401 (11.4)	0.705
Charlson Index, Mean (SD)	0.29 ± 0.76	0.31 ± 0.78	0.18 ± 0.58	< 0.001
Elixhauser Index, Mean (SD)	0.73 ± 2.66	0.8 ± 2.75	0.38 ± 2.07	< 0.001
Hernia characteristics, N (%)	–	–	–	
Recurrent hernia	1608 (7.4)	1299 (7.1)	309 (8.7)	0.001
Complicated hernia*	375 (1.7)	320 (1.8)	55 (1.6)	0.415
Type of admission, *N*(%)	–	–	–	< 0.001
Inpatient surgery	14,513 (66.6)	12,376 (67.8)	2137 (60.5)	
Outpatient surgery	7282 (33.4)	5887 (32.2)	1395 (39.5)	
Hospital volume, N (%)	–	–	–	0.144
1–20 cases/year	7279 (33.4)	6132 (33.6)	1147 (32.5)	
20–40 cases/year	7027 (32.2)	5911 (32.4)	1116 (31.6)	
40–60 cases/year	4587 (21)	3745 (20.5)	842 (23.8)	
60–80 cases/year	1595 (7.3)	1427 (7.8)	168 (4.8)	
> 80 cases/year	1307 (6)	1048 (5.7)	259 (7.3)	
Hospital length of stay (days), Mean ± SD	1.17 ± 1.87	1.2 ± 1.95	0.97 ± 1.32	< 0.001

*SD* standard deviation

Complicated hernia: hernia with obstruction or gangrene

### Factors associated with laparoscopic approach utilization

On multivariable logistic regression analysis, we observed that the main factor associated with the utilization of laparoscopy was the region of residence (*OR* 2.04, 95% *CI* 1.88–2.21). In addition, age younger than 65 years (*OR* 1.65, 95% *CI* 1.52–1.79) and recurrent inguinal hernia (*OR* 1.31, 95% *CI* 1.15–1.49) were also independently associated with the choice of laparoscopy. Comorbidities such as heart disease, renal disease, obesity, and peripheral vascular disease were negatively associated with the choice of laparoscopy (Table 3).

**Table 3:** Multivariable analysis of factors associated with the choice of laparoscopy for bilateral inguinal hernia repair

	Univariate analysis		Multivariable analysis	
	*OR* (95% *CI*)	p value	*OR* (95% *CI*)	*p* value
Region of residence*	1.99 (1.82–2.13)	< 0.001	2.04 (1.88–2.21)	< 0.001
Age < 65 years	1.81 (1.68–1.96)	< 0.001	1.65 (1.52–1.79)	< 0.001
Sex Male	1.07 (0.93–1.23)	0.371	–	–
Hospital volume > 60 cases/year	0.89 (0.79–0.98)	0.019	1.1 (0.98–1.23)	0.105
Recurrent hernia	1.25 (1.1–1.43)	0.001	1.31 (1.15–1.49)	< 0.001
Complicated hernia	0.89 (0.67–1.18)	0.415	–	–
Arterial hypertension	0.68 (0.62–0.75)	< 0.001	0.91 (0.82–1.01)	0.052
Heart disease	0.48 (0.4–0.57)	< 0.001	0.66 (0.55–0.79)	< 0.001
Chronic pulmonary disease	0.7 (0.59–0.83)	< 0.001	0.85 (0.71–1.02)	0.075
Renal disease	0.28 (0.18–0.44)	< 0.001	0.41 (0.26–0.65)	< 0.001
Liver disease	0.78 (0.56–1.08)	0.139	–	–
Diabetes mellitus	0.69 (0.59–0.79)	< 0.001	0.89 (0.77–1.05)	0.163
Obesity	0.74 (0.57–0.96)	0.024	0.71 (0.54–0.92)	0.01
Peripheral vascular disease	0.37 (0.22–0.59)	< 0.001	0.59 (0.36–0.98)	0.04
Cerebrovascular disease	0.46 (0.23–0.96)	0.037	0.66 (0.32–1.37)	0.259
Rheumatic disease	0.49 (0.28–0.84)	0.01	0.58 (0.33–1.01)	0.054

*OR* odds ratio, *CI*: confidence interval

^*^Autonomous communities of Spain with a laparoscopic rate higher than 20%

### Perioperative complications

The proportion of perioperative complications was slightly higher in the open group than in the laparoscopic group (1.8% vs. 1.1%, *p* = 0.003).

When we performed the univariate and multivariable logistic regression analysis, we observed that the approach (open or laparoscopic) was not independently associated with perioperative complications. We observed that age ≥ 65 years (*OR* 1.59, 95% *CI* 1.26–2.02), heart disease (*OR* 2.27, 95% *CI* 1.69–2.93), kidney disease (*OR* 2.56, 95% *CI* 1.66–3.88), and complicated hernia (*OR* 3.46, 95% *CI* 2.24–5.33) were independently associated with perioperative complications (Table 4).

**Table 4:** Univariate and multivariable analysis of factors associated with perioperative complications of bilateral inguinal hernia repair

	Univariate analysis		Multivariable analysis	
	*OR* (95% *CI*)	*p* value	*OR* (95% *CI*)	*p* value
Age ≥ 65	2.32 (1.87–2.87)	< 0.001	1.59 (1.26–2.02)	< 0.001
Sex Male	1.59 (0.98–2.61)	0.061	–	–
Arterial hypertension	2.25 (1.83–2.77)	< 0.001	1.25 (0.98–1.59)	0.07
Heart disease	3.73 (2.91–4.78)	< 0.001	2.27 (1.69–2.93)	< 0.001
Chronic pulmonary disease	1.93 (1.38–2.69)	< 0.001	1.19 (0.84–1.69)	0.339
Renal disease	5.23 (3.55–7.72)	< 0.001	2.56 (1.66–3.88)	< 0.001
Liver disease	2.31 (1.29–4.16)	0.005	1.78 (0.96–3.32)	0.069
Diabetes mellitus	1.97 (1.48–2.63)	< 0.001	1.18 (0.89–1.61)	0.299
Obesity	1.59 (0.93–2.74)	0.091	–	–
Peripheral vascular disease	2.66 (1.44–4.9)	0.002	1.15 (0.61–2.18)	0.664
Cerebrovascular disease	3.88 (1.69–8.91)	0.001	2.33 (0.99–5.42)	0.051
Rheumatic disease	2.65 (1.24–5.69)	0.012	1.84 (0.84–4.04)	0.126
Alcohol abuse	1.88 (1.07–3.3)	0.028	1.36 (0.74–2.46)	0.323
Tobacco use	1.29 (0.96–1.73)	0.088	–	–
Recurrent hernia	1.63 (1.18–2.25)	0.003	1.39 (0.99–1.94)	0.053
Complicated hernia	4.38 (2.88–6.66)	< 0.001	3.46 (2.24–5.33)	< 0.001
Open approach	1.65 (1.18–2.3)	0.003	1.36 (0.97–1.91)	0.076

*OR*: odds ratio, *CI*: confidence interval

## Discussion

The proportion of laparoscopic BIHR has increased in recent years in Spain; however, it is still low. The likelihood of having a laparoscopic procedure seems to depend on whether the patient has a hospital or surgeons who perform many laparoscopic procedures near their residence. The approach (open or laparoscopic) for BIHR was not associated with increased perioperative complications.

The BIHR has changed in recent years. Initially, the treatment was performed through two sequential repair surgeries because a higher rate of recurrence and complications was described when performing simultaneous repairs [23–25]. However, good results were later described with a simultaneous repair, which avoids double anesthesia, a double limitation of physical activity, and a longer period of sick leave [26]. Therefore, it is now recommended that simultaneous repair should be the standard technique for bilateral hernias [14].

Another change in the BIHR has occurred in the type of approach. The development of laparoscopic techniques offers a new alternative to conventional treatment. The advantages include reduced postoperative pain, lower postoperative complications, shorter hospital stays, and shorter recovery [8, 9, 27]. Nowadays, the recommendation of the international clinical guidelines, from a socioeconomic perspective, is to perform the repair of bilateral inguinal hernias by laparoscopic approach [11–15].

However, despite the advantages described and recommendations from international surgical societies, laparoscopic repair has been slow to gain acceptance in Spain [8]. We observed a significant increase in the use of the laparoscopic approach for BIHR from 12% in 2016 to 23% in 2019. However, it remains low compared to other countries where the laparoscopic approach is used in more than half of the cases [19, 28]. In our study, only 8% of the hospitals used laparoscopy in more than half of the BIHRs, and 40% only performed open surgery.

Some factors contributing to the low rate of laparoscopic bilateral hernia repair in Spain are structural and depend on the national health system and individual hospital organizations. Hernia repair is a highly prevalent procedure, with a significant waiting list in some areas of the country. The health system often encourages the number of procedures and the lowest cost per session over quality [29].

When analyzing the factors associated with the choice of approach for the BIHR, we observe that the main one is the region of residence. There were important differences in the proportion of laparoscopic BIHR according to the region of residence, ranging from 0 to 46%. Only four Autonomous Communities in the country have rates greater than 20% of BIHR by laparoscopy, which can be explained by the greater presence of hospitals with high use of laparoscopy in these regions. In Spain, each Autonomous community or region is directly responsible for planning, managing, and administering health matters. This decentralized healthcare management system may explain differences in the use of laparoscopy between regions.

We also observed that performing a higher number of BIHR per year is not related to the decision to perform them laparoscopically. A large surgical waiting list and a health care system that often incentivizes the number of procedures and lower cost over quality [29] may explain why hospitals with large numbers of procedures have not transitioned to laparoscopy.

In Spain, the national health system includes public and private state-contracted hospitals with similar healthcare resources throughout the country. The use of the laparoscopic approach in each hospital seems to depend on local incentives or decisions of surgeons or surgical teams.

The learning curve is essential for achieving good results [13, 30] and continues to be a reason for the slow acceptance of laparoscopy. Access to adequate training for surgeons and residents through theoretical-practical courses and training in simulators would allow the learning curve to cease to be an obstacle to the use of laparoscopy in BIHR [31, 32]. A structured and systematized training process allows a safe transition to laparoscopy, even in small hospitals [33]. In our study, we observed a significant increase in the use of laparoscopic repair in recent years, probably due to greater access to training for new technologies and resident training programs by professional associations.

Another reason that limits the choice of laparoscopic approach by surgical teams is cost. From a hospital perspective, open BIHR is more cost-effective. However, from a socioeconomic perspective, a laparoscopic procedure for BIHR is the most cost-effective approach, especially for patients in the labor market [13]. Laparoscopic BIHR has low morbidity, shorter recovery, and faster return to work time [34, 35]. In this line, a recent randomized trial showed that laparoscopic TAPP repair for bilateral inguinal hernia represents a cost-effective procedure compared to open repair [36].

When comparing the results of open and laparoscopic BIHR, we observed a shorter mean hospital stay in the laparoscopic group. These results are like previous studies [37, 38]. This shorter hospital stay can be explained by the lower postoperative pain described in patients undergoing laparoscopic repair [35]. Our study also observed more outpatient surgeries in the laparoscopic group. Worldwide, there is a clear increase in the percentage of inguinal hernia repairs performed as outpatient surgery [39], and its use is recommended regardless of the technique [13]. The greater use of laparoscopic surgery in BIHR would probably increase the percentage of ambulatory surgeries and therefore decrease hospital costs.

In recent meta-analyses, no differences have been found in the incidence of postoperative complications of open and laparoscopic unilateral inguinal hernia repair [40, 41]. However, studies on bilateral inguinal hernias have reported that postoperative complications of open repair were greater than those of laparoscopic repair [36, 42]. Our study found a higher incidence of perioperative complications in the open approach group. However, when we performed a multivariable analysis, the type of approach was not associated with perioperative complications. This could be because the patients with the open repair were older and had more comorbidity. However, these data should be taken with caution. The low number of complications detected may be due to incomplete coding of perioperative complications.

As it is a clinical-administrative database that only describes data during hospital admission, we don’t have data on post-admission complications, chronic pain, or recurrence. Literature reports a lower incidence of chronic pain or less severity of pain in laparoscopic inguinal hernia repairs [40, 43]. Regarding recurrence, the long-term comparative results between open and laparoscopic repair show no significant differences [42, 44, 45].

The limitation of our study is the potential underreporting of information because the hospital discharge report may be incomplete or poorly recorded by the technical-administrative staff. In addition, the data in this clinical-administrative database do not include detailed information on the surgical technique used in each case or the evolution of the patient after the hospital stay, and it does not provide information on complications such as chronic pain or recurrence.

The strength of our study is its large sample size, which provides strong statistical power. Since it records almost all Spanish National Health System hospital admissions, it reinforces its external validity. The RAE-CMBD database has several internal audit mechanisms and has proven useful for health research [20, 46, 47].

## Conclusion

The choice of the laparoscopic approach for BIHR in Spain is still low despite its significant increase in recent years. The likelihood of having a laparoscopic procedure seems to depend on whether the patient has a hospital or surgeons who perform many laparoscopic procedures near their residence. The type of approach chosen was not independently associated with the development of perioperative complications. More efforts are needed to increase laparoscopic use in patients with bilateral inguinal hernias. Training residents and surgeons in laparoscopic techniques and knowledge of the socioeconomic benefits of laparoscopy could increase its use in BIHR and help follow the recommendations of international guidelines.

## Supplementary Information

Below is the link to the electronic supplementary material.

Supplementary file1 (DOCX 16 KB)

## Data Availability

The datasets used and analyzed during the current study are available from the corresponding author on reasonable request.

## References

[1] Kurzer M, Kark A, Hussain T (2007). Inguinal hernia repair. J Perioper Pract..

[2] McCormack K, Wake BL, Fraser C, Vale L, Perez J, Grant A (2005). Transabdominal pre-peritoneal (TAPP) versus totally extraperitoneal (TEP) laparoscopic techniques for inguinal hernia repair: a systematic review. Hernia.

[3] Kingsnorth A, LeBlanc K (2003). Hernias: inguinal and incisional. Lancet.

[4] Arregui ME, Davis CJ, Yucel O, Nagan RF (1992). Laparoscopic mesh repair of inguinal hernia using a preperitoneal approach: a preliminary report. Surg Laparosc Endosc.

[5] McKernan JB, Laws HL (1993). Laparoscopic repair of inguinal hernias using a totally extraperitoneal prosthetic approach. Surg Endosc.

[6] Claus CMP, Rocha GM, Campos ACL, Bonin EA, Dimbarre D, Loureiro MP, Coelho JCU (2016). Prospective, randomized and controlled study of mesh displacement after laparoscopic inguinal repair: fixation versus no fixation of mesh. Surg Endosc.

[7] Ross SW, Oommen B, Kim M, Walters AL, Augenstein VA, Heniford BT, Todd Heniford B (2015). Tacks, staples, or suture: method of peritoneal closure in laparoscopic transabdominal preperitoneal inguinal hernia repair effects early quality of life. Surg Endosc.

[8] Feliu-Palà X, Martín-Gómez M, Morales-Conde S, Fernández-Sallent E (2001). The impact of the surgeon’s experience on the results of laparoscopic hernia repair. Surg Endosc.

[9] Schmedt CG, Sauerland S, Bittner R (2005). Comparison of endoscopic procedures vs Lichtenstein and other open mesh techniques for inguinal hernia repair: a meta-analysis of randomized controlled trials. Surg Endosc.

[10] Bittner RR, Felix EL (2021). History of inguinal hernia repair, laparoendoscopic techniques, implementation in surgical praxis, and future perspectives: considerations of two pioneers. Int J Abdom Wall Hernia Surg.

[11] Bittner R, Montgomery MA, Arregui E, Bansal V, Bingener J, Bisgaard T, Buhck H, Dudai M, Ferzli GS, Fitzgibbons RJ, Fortelny RH, Grimes KL, Klinge U, Köckerling F, Koeckerling F, Kumar S, Kukleta J, Lomanto D, Misra MC, Morales-Conde S, Reinpold W, Rosenberg J, Singh K, Timoney M, Weyhe D, Chowbey P, International Endohernia Society (2015). Update of guidelines on laparoscopic (TAPP) and endoscopic (TEP) treatment of inguinal hernia (International Endohernia Society). Surg Endosc.

[12] Poelman MM, van den Heuvel B, Deelder JD, Abis GSA, Beudeker N, Bittner RR, Campanelli G, van Dam D, Dwars BJ, Eker HH, Fingerhut A, Khatkov I, Koeckerling F, Kukleta JF, Miserez M, Montgomery A, Munoz Brands RM, Morales Conde S, Muysoms FE, Soltes M, Tromp W, Yavuz Y, Bonjer HJ (2013). EAES Consensus Development Conference on endoscopic repair of groin hernias. Surg Endosc..

[13] Simons MP, Aufenacker T, Bay-Nielsen M, Bouillot JL, Campanelli G, Conze J, de Lange D, Fortelny R, Heikkinen T, Kingsnorth A, Kukleta J, Morales-Conde S, Nordin P, Schumpelick V, Smedberg S, Smietanski M, Weber G, Miserez M (2009). European Hernia Society guidelines on the treatment of inguinal hernia in adult patients. Hernia.

[14] HerniaSurge Group (2018). International guidelines for groin hernia management. Hernia.

[15] Bittner R, Arregui ME, Bisgaard T, Dudai M, Ferzli GS, Fitzgibbons RJ, Fortelny RH, Klinge U, Kockerling F, Kuhry E, Kukleta J, Lomanto D, Misra MC, Montgomery A, Morales-Conde S, Reinpold W, Rosenberg J, Sauerland S, Schug-Pass C, Singh K, Timoney M, Weyhe D, Chowbey P (2011). Guidelines for laparoscopic (TAPP) and endoscopic (TEP) treatment of inguinal hernia [International Endohernia Society (IEHS)]. Surg Endosc.

[16] Smink DS, Paquette IM, Finlayson SRG (2009). Utilization of laparoscopic and open inguinal hernia repair: a population-based analysis. J Laparoendosc Adv Surg Tech A.

[17] Madion M, Goldblatt MI, Gould JC, Higgins RM (2021). Ten-year trends in minimally invasive hernia repair: a NSQIP database review. Surg Endosc.

[18] AlMarzooqi R, Tish S, Huang L-C, Prabhu A, Rosen M (2019). Review of inguinal hernia repair techniques within the Americas Hernia Society Quality Collaborative. Hernia.

[19] Palser TR, Swift S, Williams RN, Bowrey DJ, Beckingham IJ (2019). Variation in outcomes and use of laparoscopy in elective inguinal hernia repair. BJS Open.

[20] Guillaumes S, Hoyuela C, Hidalgo NJ, Juvany M, Bachero I, Ardid J, Martrat A, Trias M (2021). Inguinal hernia repair in Spain. A population-based study of 263,283 patients: factors associated with the choice of laparoscopic approach. Hernia.

[21] Ministerio de Sanidad C y BS (2021) Registro de Actividad de Atención Especializada del Conjunto Mínimo Básico de Datos (RAE-CMBD). https://www.mscbs.gob.es/estadEstudios/estadisticas/estadisticas/estMinisterio/SolicitudCMBD.htm

[22] Quan H, Sundararajan V, Halfon P, Fong A, Burnand B, Luthi J-C, Saunders LD, Beck CA, Feasby TE, Ghali WA (2005). Coding algorithms for defining comorbidities in ICD-9-CM and ICD-10 administrative data. Med Care.

[23] Berndsen F, Petersson U, Montgomery A (2001). Endoscopic repair of bilateral inguinal hernias–short and late outcome. Hernia.

[24] Wauschkuhn CA, Schwarz J, Boekeler U, Bittner R (2010). Laparoscopic inguinal hernia repair: gold standard in bilateral hernia repair? results of more than 2800 patients in comparison to literature. Surg Endosc.

[25] Jacob DA, Hackl JA, Bittner R, Kraft B, Köckerling F (2015). Perioperative outcome of unilateral versus bilateral inguinal hernia repairs in TAPP technique: analysis of 15,176 cases from the Herniamed Registry. Surg Endosc.

[26] Pfeffer F, Riediger H, Küfner Lein R, Hopt UT (2008). Repair of bilateral inguinal hernias–sequential or simultaneous?. Zentralbl Chir.

[27] Feliu X, Clavería R, Besora P, Camps J, Fernández-Sallent E, Viñas X, Abad JM (2011). Bilateral inguinal hernia repair: laparoscopic or open approach?. Hernia.

[28] Zendejas B, Ramirez T, Jones T, Kuchena A, Martinez J, Ali SM, Lohse CM, Farley DR (2012). Trends in the utilization of inguinal hernia repair techniques: a population-based study. Am J Surg..

[29] Feliu Palà X (2015). Laparoscopic surgery of the abdominal wall: Why has it not been implemented like other laparoscopic procedures?. Cir Esp.

[30] Lovisetto F, Zonta S, Rota E, Mazzilli M, Bardone M, Bottero L, Faillace G, Longoni M (2007). Use of human fibrin glue (Tissucol) versus staples for mesh fixation in laparoscopic transabdominal preperitoneal hernioplasty: a prospective, randomized study. Ann Surg.

[31] Ivakhov G, Kolygin A, Titkova S, Anurov M, Sazhin A (2020). Development and evaluation of a novel simulation model for transabdominal preperitoneal (TAPP) inguinal hernia repair. Hernia.

[32] Köckerling F, Sheen AJ, Berrevoet F, Campanelli G, Cuccurullo D, Fortelny R, Friis-Andersen H, Gillion JF, Gorjanc J, Kopelman D, Lopez-Cano M, Morales-Conde S, Österberg J, Reinpold W, Simmermacher RKJ, Smietanski M, Weyhe D, Simons MP (2019). The reality of general surgery training and increased complexity of abdominal wall hernia surgery. Hernia.

[33] Hidalgo NJ, Bachero I, Hoyuela C, Juvany M, Ardid J, Martrat A, Guillaumes S (2022). The transition from open to laparoscopic surgery for bilateral inguinal hernia repair: how we did it. Langenbecks Arch Surg.

[34] Sarli L, Iusco DR, Sansebastiano G, Costi R (2001). Simultaneous repair of bilateral inguinal hernias: a prospective, randomized study of open, tension-free versus laparoscopic approach. Surg Laparosc Endosc Percutan Tech.

[35] Mahon D, Decadt B, Rhodes M (2003). Prospective randomized trial of laparoscopic (transabdominal preperitoneal) vs open (mesh) repair for bilateral and recurrent inguinal hernia. Surg Endosc.

[36] Ielpo B, Nuñez-Alfonsel J, Duran H, Diaz E, Fabra I, Caruso R, Malavé L, Ferri V, Barzola E, Quijano Y, Vicente E (2018). Cost-effectiveness of randomized study of laparoscopic versus open bilateral inguinal hernia repair. Ann Surg.

[37] Vale L, Ludbrook A, Grant A (2003). Assessing the costs and consequences of laparoscopic vs. open methods of groin hernia repair: a systematic review. Surg Endosc.

[38] Ielpo B, Duran H, Diaz E, Fabra I, Caruso R, Malavé L, Ferri V, Lazzaro S, Kalivaci D, Quijano Y, Vicente E (2018). A prospective randomized study comparing laparoscopic transabdominal preperitoneal (TAPP) versus Lichtenstein repair for bilateral inguinal hernias. Am J Surg.

[39] de Lathouwer C, Poullier, (2000). How much ambulatory surgery in the World in 1996–1997 and trends?. Ambul Surg.

[40] Scheuermann U, Niebisch S, Lyros O, Jansen-Winkeln B, Gockel I (2017). Transabdominal Preperitoneal (TAPP) versus Lichtenstein operation for primary inguinal hernia repair—a systematic review and meta-analysis of randomized controlled trials. BMC Surg.

[41] Wu JJ, Way JA, Eslick GD, Cox MR (2018). Transabdominal pre-peritoneal versus open repair for primary unilateral inguinal hernia: a meta-analysis. World J Surg.

[42] Elmessiry MM, Gebaly AA (2020). Laparoscopic versus open mesh repair of bilateral primary inguinal hernia: a three-armed randomized controlled trial. Ann Med Surg (Lond).

[43] Bignell M, Partridge G, Mahon D, Rhodes M (2012). Prospective randomized trial of laparoscopic (transabdominal preperitoneal-TAPP) versus open (mesh) repair for bilateral and recurrent inguinal hernia: incidence of chronic groin pain and impact on quality of life: results of 10 year follow-up. Hernia.

[44] O’Dwyer PJ (2004). Current status of the debate on laparoscopic hernia repair. Br Med Bull.

[45] Hallén M, Bergenfelz A, Westerdahl J (2008). Laparoscopic extraperitoneal inguinal hernia repair versus open mesh repair: long-term follow-up of a randomized controlled trial. Surgery.

[46] Pedraza-Serrano F, Jiménez-García R, López-de-Andrés A, Hernández-Barrera V, Esteban-Hernández J, Sánchez-Muñoz G, Puente-Maestu L, de-Miguel-Díez J, (2018). Comorbidities and risk of mortality among hospitalized patients with idiopathic pulmonary fibrosis in Spain from 2002 to 2014. Respir Med.

[47] Ribera A, Marsal JR, Ferreira-González I, Cascant P, Pons JMV, Mitjavila F, Salas T, Permanyer-Miralda G (2008). Predicting in-hospital mortality with coronary bypass surgery using hospital discharge data: comparison with a prospective observational study. Rev Esp Cardiol.

